# Crocin mitigated cognitive impairment and brain molecular alterations induced by different intensities of prenatal hypoxia in neonatal rats

**DOI:** 10.1002/brb3.2078

**Published:** 2021-02-15

**Authors:** Zohreh Ghotbeddin, Mohammad Reza Tabandeh, Mahdi Pourmahdi Borujeni, Fahimeh Fahimi Truski, Mohammad Reza Zalaki Ghorbani Pour, Leila Tabrizian

**Affiliations:** ^1^ Division of Physiology Department of Basic Sciences Faculty of Veterinary Medicine Shahid Chamran University of Ahvaz Ahvaz Iran; ^2^ Stem Cell and Transgenic Technology Research Center Shahid Chamran University of Ahvaz Ahvaz Iran; ^3^ Division of Biochemistry and Molecular Biology Department of Basic Sciences Faculty of Veterinary Medicine Shahid Chamran University of Ahvaz Ahvaz Iran; ^4^ Department of Food Hygiene Faculty of Veterinary Medicine Shahid Chamran University of Ahvaz Ahvaz Iran

**Keywords:** cognitive behavior, crocin, hypoxia, molecular changes

## Abstract

**Introduction:**

Brain hypoxia has important role to the onset and progression of sporadic form of Alzheimer disease via expression of hypoxia‐inducible factor‐1 (HIF‐1). Crocin by anti‐amyloidogenic property inhibits β‐amyloid formation. However, the molecular mechanism associated with anti‐amyloidogenic activity of crocin is unknown. So, the present study was designed to investigate the effect of crocin on cognitive behavior and expression of HIF‐1α and β‐secretase (BACE1) genes in the brain of neonate rats following different intensities of hypoxia during pregnancy.

**Material and methods:**

Pregnant female rats were divided into six groups including sham, control crocin treated (CC), hypoxia with three different intensities (H1–H3), and most intense of hypoxic group treated with crocin (H3C) (30 mg/kg; i.p) at P14. Hypoxia induced on the 20th day of pregnancy. Animals in sham and CC were put in hypoxia chamber at the same time of hypoxia group without any hypoxia induction. Morris water maze (MWM) and qRT‐PCR were used to evaluate the cognitive behavior and mRNA levels of BACE1 and HIF‐1α genes in the brain tissues.

**Results:**

Animal under 7% O_2_ + 93% N_2_ condition for 3 hr showed the highest cognitive behavior impairment and upregulated HIF‐1α and BACE1 mRNA in brains of offspring (*p* < .001). Crocin treatment improved memory impairment and attenuated the gene expression of HIF‐1α and BACE1 in the brains of neonate rat.

**Conclusions:**

It was concluded that crocin has beneficial effects on the brain of neonate rats under gestational hypoxia by improvement of memory impairment and molecular alteration related to hypoxia.

## Graphical abstract

Since over expression of HIF‐1α and BACE1 genes related to cognitive behavior impairment and duration, intensity of oxygen deprivation is an important factors in brain dysfunction caused by hypoxia, on the other hand; many studies have pointed the effective role of crocin in learning and memory improvement. Without addressing the molecular mechanism, in this study we evaluated crocin effect on cognitive task and expression of HIF‐1α and BACE1 genes in offspring's brains which influenced by three intensities of maternal hypoxia. Our main research findings showed that hypoxia with 93% N_2_ and 7% O_2_ intensity for 3 hr had the most destructive effect on cognitive behavior and mRNA level of genes related to cognition in rat's offspring. Crocin had a protective effect by inhibiting the molecular pathway to β‐amyloid formation and cognitive impairment.

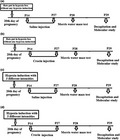

## 1 INTRODUCTION

Hypoxia is considered as an environmental factor in the onset of various neurodegenerative diseases (Getahun et al., 2013). Hypoxia usually occurs when there is not enough oxygen for metabolic functions; in other words, when arterial oxygen pressure is less than 65 mmHg (Koeppen & Stanton, 2009). Experimental and clinical studies showed that hypoxia during pregnancy can change function of the hippocampus and affect the behavior of the infants until the end of life (Getahun et al., 2013). It has been shown that peoples who have severe hypoxia or ischemia are more likely to suffer from memory problem (Desmond et al., 2002). Pregnancy hypoxia not only increases the risk of abortion, but also results in neuronal development disorders in childhood (Depino, 2015). Duration, intensity of oxygen deprivation, and age of fetus are an important factors in temporary brain dysfunction or permanent brain injury caused by hypoxia (Golan et al., 2004). There are a lot of biomarker which are affected and changed after hypoxia. These changes are initiated during an acute event with a loss of high‐energy phosphate compounds, intracellular acidosis followed by excessive extracellular glutamate accumulation, accumulation of calcium inner the cell, free radical generation as well as activation of inflammatory mediators that may extend after the initial process and lead to neuronal death (Perlman, 2007). Mostly, hypoxic responses at the molecular level are managed by hypoxia‐inducible transcription factor‐1 (HIF‐1). The HIF complex is a heterodimer consisting of two subunits, HIF‐1β subunit (also known as the aryl hydrocarbon receptor nuclear translocation, ARNT) and a HIF‐1α oxygen‐regulated subunit. Normoxic conditions result in HIF‐1α degradation (Semenza, 1998). HIF‐1α level is low in normal and physiologic condition, but in a low oxygen environment it dramatically increases (Wenger & Gassmann, 1997).

The pathological signs of AD are characterized by formation and accumulation of β‐amyloid (Aβ) peptide as plaque in extracellular and deposition of Tau protein as neurofibrillary tangles as well as loss of neurons and synapses (Mondragón‐Rodríguez et al., 2012; Nisbet et al., 2015). These markers are usually formed in areas associated with learning and memory. The formation of two Aβ isoforms (Aβ_40_ and Aβ_42_) is due to the cleavage of amyloid precursor protein (APP) by β‐secretase (b‐site amyloid precursor protein cleavage enzyme, BACE) and then by the γ‐secretase complex (with presenilin1) (Wang et al., 2015). β‐amyloid peptide produced by cleavage of amyloid precursor protein through secretases is responsible for death of neurons and dementia in Alzheimer's disease. Recent data suggest that hypoxic condition such as ischemia changes the expression of secretases and promotes overproduction and aggregation of β‐amyloid peptide, resulting in impairment of brain function (Nalivaeva et al., 2004). Studies using knockout mice demonstrated that BACE‐1 is necessary for neuronal Aβ peptide generation, making it a particularly good agent for the generation of inhibitors that lower Aβ levels (Venugopal et al., 2006). Hypoxia increases the activity of BACE1 by induction of expression of HIF‐1α. The mRNA and protein levels of BACE1 are increased by overexpression of HIF‐1α (Zhang et al., 2007). Enhanced BACE1 activity following hypoxia can also increase the production of β‐amyloid (βA_42_ and βA_40_) from APP (Tamagno, Bardini, et al., 2012; Tamagno et al., 2012).

In recent years, the usage of effective herbal compounds to prevent or decrease the side effects of neurodegenerative diseases is considered. Although the antioxidant effects of these compounds have been proven, but the molecular effect of them remains unknown. Crocin is one of the most important herbal compounds which is considered clinically in disease associated with memory impairment. Crocin as a water‐soluble carotenoid is an active constituent of Saffron (*Crocus sativus* L.). Previous studies have shown that Saffron extract could improve the learning and cognitive function and could distribute oxygen in different tissues (Georgiadou et al., 2014; Naghizadeh et al., 2013). Results of recent investigations have shown that crocin has an anti‐amyloidogenic property by inhibition of β‐amyloid formation and destruction of accumulated β‐amyloid (Huang & Jiang, 2009). Crocin also antagonizes memory impairment following the injection of Streptozotocin (STZ) in the Alzheimer's sporadic model (Naghizadeh et al., 2013). Since over expression of HIF‐1α and BACE1 genes related to cognitive behavior impairment and duration, intensity of oxygen deprivation is an important factors in brain dysfunction caused by hypoxia, on the other hand; many studies have pointed the effective role of crocin in learning and memory improvement, without addressing the molecular mechanism, in this study we evaluated crocin effect on cognitive task and expression of HIF‐1α and BACE1 genes in offspring's brains which influenced by three intensities of maternal hypoxia.

## 2 METHODS AND MATERIALS

### 2.1 Animals and experimental groups

In this experimental study, nine female and three male rats were used for mating in each experimental group. All rats were placed in cages under standard laboratory conditions including ambient temperature 23–25°C, 12 hr light/dark alternate cycle with lights on between 7:00 a.m. and 7:00 p.m., and free access to food and water. Mating was achieved by placing three females and one male in a cage overnight, and successful mating was confirmed by the presence of sperm in vaginal cytology on the following days. That day was considered as day 0 of gestation.

**FIGURE 1:**
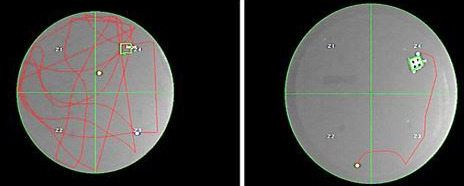
Captured images of rat movement in the Morris water maze by video tracking software

Schematic diagram of our procedure and grouping is shown in Figure 1. Hypoxic exposure was conducted in a Plexiglas chamber equipped with a small fan to provide forced circulation and almost instantaneous homogenization of gases within the chamber. The oxygen content was adjusted by a nitrogen/compressed air gas delivery system that mixes the nitrogen with room air using a compact oxygen controller (TajhizSanat). Temperature, lighting, and humidity were maintained at standard vivarium levels, and the chamber was opened for 5–10 min biweekly for routine care. The pregnant rats were randomly divided into six experimental groups as follows: sham, hypoxia with three different intensities: 10% O_2_ and 90% N_2_ for 1 hr (H1), 7% O_2_ and 93% N_2_ for 1 hr (H2), 7% O_2_ and 93% N_2_ for 3 hr (H3) (Ghotbeddin et al., 2018), H3 group that neonates were treated with crocin (H3C) and a control crocin treated (CC) group without induction of prenatal hypoxia. Animals in sham were put in hypoxia chamber on the 20th day of pregnancy without any hypoxia induction and had access to complete air flow. HC groups were put in hypoxia chamber with the same intensity similar to hypoxia groups, and all newborn animals were treated i.p with crocin (30 mg/kg) from P14 to P27. Animals in CC group were put in hypoxia chamber without induction of hypoxia, and all neonates were treated i.p with crocin (30 mg/kg) from P14 to P27. Cognitive behavior was tested in all groups by Morris water maze at P28, and we used five to seven pups in each behavioral and molecular experiment (Figure 1).

All stages of the experiment were designed and executed in accordance with the instructions of the ethics committee of laboratory animals of the Shahid Chamran University of Ahvaz. All animals used were cared for according to the guide for the care and use of laboratory animals by the national academy of sciences (National Institutes of Health publication No. 86‐23).

### 2.2 Morris water maze test

**FIGURE 2:**
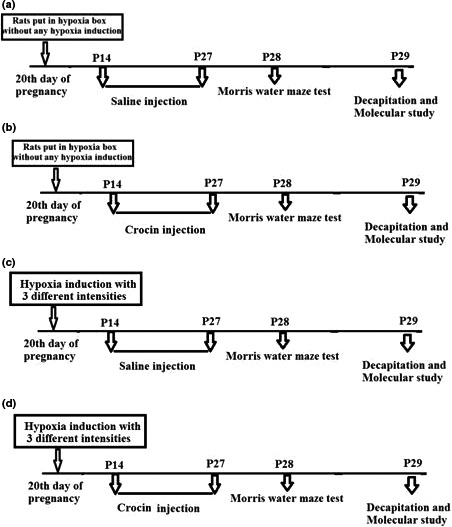
Schematic diagram of the experimental procedures in different experimental groups including sham (a), crocin treated control; CC (b), hypoxia; H1‐H3 (c) and hypoxia treated with crocin; HC

In all groups, Morris water maze (MWM) test was used to evaluate animal's cognitive behavior (spatial memory). MWM consisted of a dark circular tank with 160 cm diameter and 60 cm high which is filled up to 35 cm with a water temperature of 25 ± 2. The tank is divided into four quadrants of equal circles (north, south, east, and west), and one point was considered for dropping animals. An invisible platform with 10 cm diameter was located in the center of the target quadrant, 1/5 cm below the surface of the water. Different geometric shapes as visual clues were installed on the walls of the tank. Animal's performance through a video tracking camera attached to the top of the maze was transmitted to the computer and software to record required parameters including traveled distance and latency time to find the platform. Spatial learning was examined in three blocks with 30 s intervals. Each block consisted of four trials, and in each trials, rat released to water from one of the quarters circles which selected by device randomly and animal head located toward wall of the maze; it takes a maximum of 60 s to find and rest on the hidden platform below the surface of the water using visual clues. If the animal could not find the platform in 60 s, the researcher guided the animal to the platform by hand and rest for 30–35 s on it. Then, animals rested again for 30–35 s in a box under the lamp. The next trials were done the same by releasing the animal from other quadrants. Traveled distance and latency time to find the hidden platform in these three blocks were considered as criteria for animal's spatial memory. A single probe trial was given 2 hr after the last training trial to test the spatial memory in the water maze. In this trial, the platform was removed and rat was allowed to swim for 60 s. Swimming speed and traveled distance in the target quadrant were analyzed as the measure of spatial memory retention (Figure 2) (Frick et al., 2000).

### 2.3 RT‐PCR method

To determine the effect of prenatal hypoxia and protective effect of crocin against it, gene expression of HIF1α and BACE1 was analyzed in brain of neonates at P29 using qRT‐PCR analysis. After behavioral test, offspring were anesthetized by ketamine (100 mg/kg) and xylazine (10 mg/kg) and their brain were removed quickly and kept at −70°C until molecular experiments. Total RNA was isolated from brain using RNX™ isolation kit according to the manufacturer's procedure (Sinaclone) using of 100 mg of tissue. Optical density (*A*_260_/*A*_280_ and *A*_260_/*A*_230_) and concentration were measured using Eppendorf µCuvette G1.0 microvolume measuring cell (Eppendorf BioPhotometer D30, Eppendorf). RNA samples with a ratio more than 1.8 were used for cDNA Synthesis. Total RNA was converted to cDNA by using random hexamer primers and YTA cDNA synthesis kit (Yektatajhiz). For qRT‐PCR, three replicates per sample were amplified and analyzed using a Roche Light Cycler. Specific sets of primers (Bioneer) designed for this study were as follows: HIF1α (GenBank: NM_024359): 5′‐GTACCCTAACTAGCCGAGGAAGAA‐3′ and 5′‐GTGAATGTGGCCTGTGCAGT‐3′, GAPDH (GenBank: NM_NM‐001034055): 5′‐CTCATCTACCTCTCCATCGTCTG‐3′ and 5′‐CCTGCTCTTGTCTGCCGGTGCTTG‐3′ and BACE1 (GenBank: NM_019204): 5′‐GCTGCAGTCAAGTCCATCAA‐3′ and 5′‐ATTGCTGAGGAAGGATGGTG‐3′. Reactions were carried out in a 12.5 μl volume containing 6.25 μl qPCR™ Green Master Kit for SYBR Green I^®^ (Yektatajhiz), 0.25 μl of each primer (200 nM), 3 μl cDNA (100 ng), and 2.25 μl nuclease‐free water. The PCR protocol used consisted of a 5 min denaturation at 94°C followed by 45 cycles of 94°C for 15 s, 60°C for 15 s, and 72°C for 30 s. Two separate reactions without cDNA or with RNA were performed in parallel as controls. The relative gene expression levels were determined using the comparative threshold cycle (2^−ΔΔCT^) method and Light cycler 96^®^ software. Validation of assay to check that the primer for the BACE1 and HIF1α had similar amplification efficiencies was performed as described previously. All qPCR analysis was performed according to The Minimum Information for Publication of Quantitative Real‐Time PCR Experiments (MIQE) guideline (Bustin et al., 2009).

### 2.4 Statistical analysis

The values are presented as the mean ± *SEM*. Normality of the data was assessed using Kolmogrov–Smirnov test. Parameters in training phase of Morris water maze test were calculated by two‐way ANOVAs. Another parameters in the probe phase of the Morris water maze and molecular assay were analyzed by one‐way ANOVA followed by Tukey post hoc. The level of significance was set at the *p* < .05 for all statistical tests. All statistical assessments and graph plotting were performed using GraphPad Prism 8 software.

## 3 RESULTS

### 3.1 Spatial memory assay using the Morris water maze test

Spatial memory measurement included two stages: learning and probe. Traveled distance (TD) and latency time (LT) to find the platform were measured in the learning stage. Swimming speed (SS) and traveled distance (TD) in the target quadrant were assessed in the probe stage. Initially, all hypoxia groups with three different intensity (H1, H2, and H3) compared with sham group to find the most destructive intensity of hypoxia. After that, to find the effect of crocin injection in offspring which was affected by prenatal hypoxia, the H3 was compared with H3C, control crocin treated (CC) and sham groups.

#### 3.1.1 Traveled distance and latency time to find the hidden platform

**FIGURE 3:**
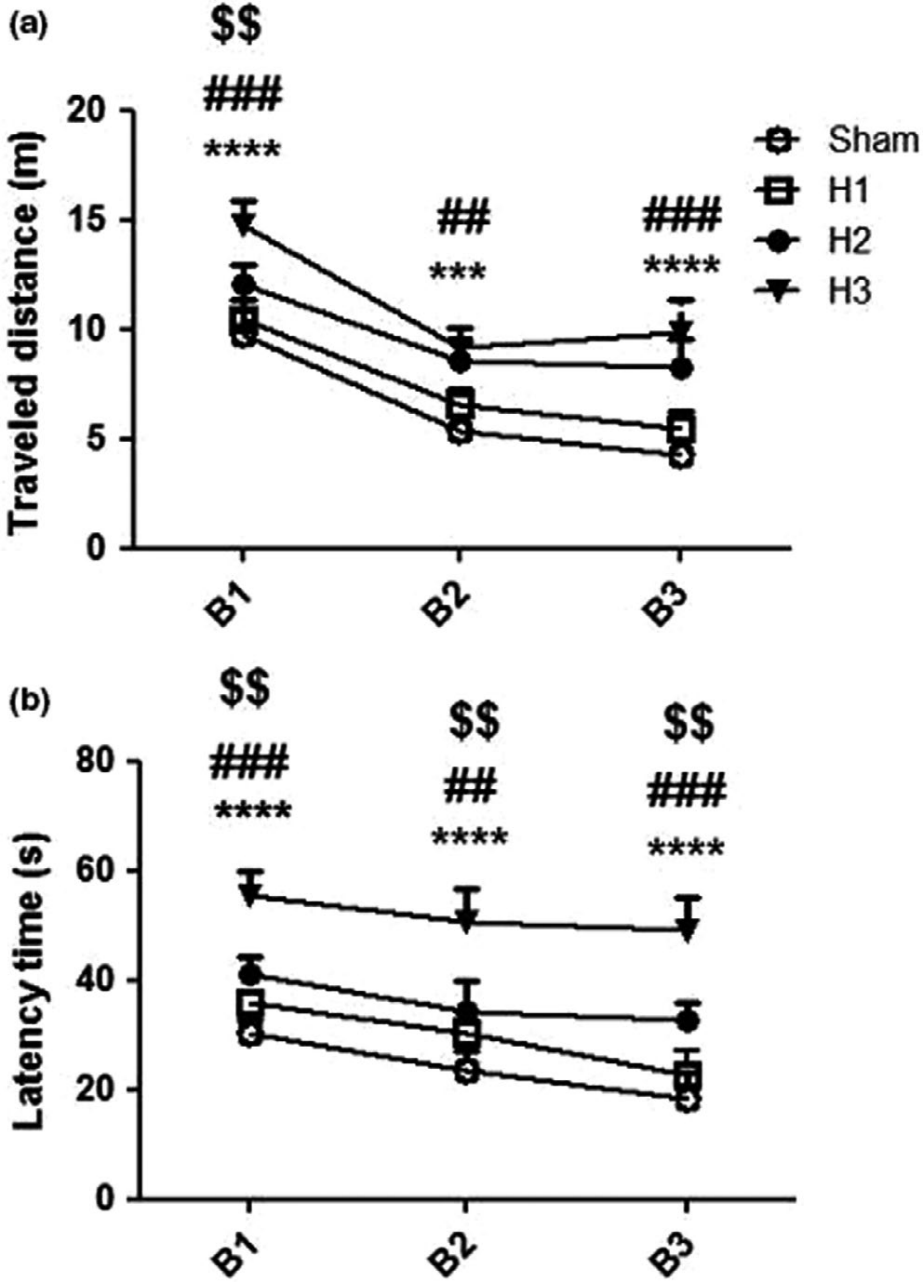
Traveled distanced (a) and latency time (b) to find the platform was significantly increased in H3 compared to the sham and other hypoxia groups (H1 and H2). B1, B2, and B3; three blocks of training stage in Morris water maze test. ***p < .001, ****p < .0001 as compared to the sham group. ##*p* < .01, ###*p* < .001 in comparison with the H1 group and $$*p* < .01 as compared to the H2 rats. Data presented as mean ± *SEM* (*n* = 7)

**FIGURE 4:**
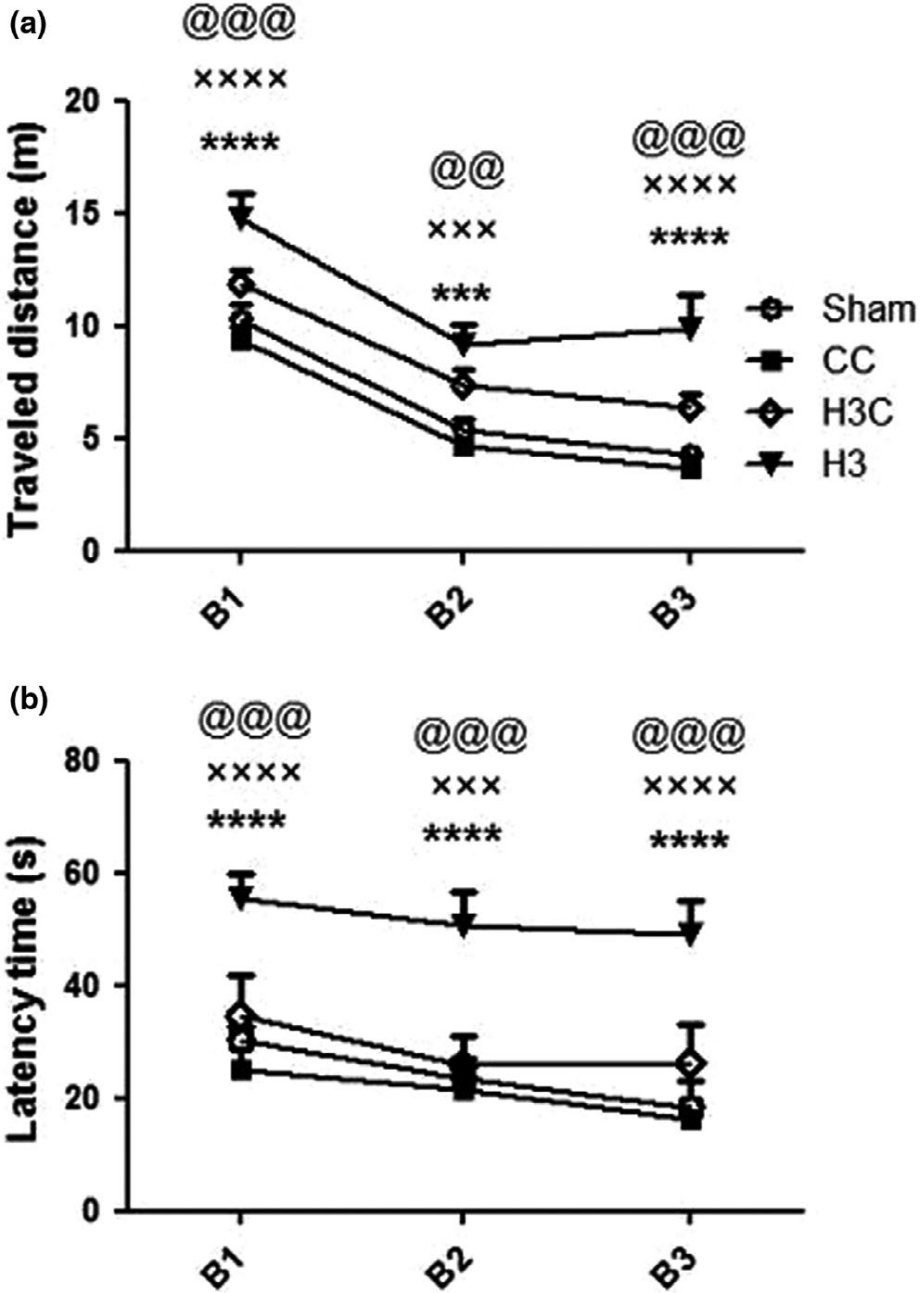
Traveled distanced (a) and latency time (b) to find the platform was significantly increased in H3 compared to the sham, CC, and H3C groups. H3: hypoxia 3; CC: control crocin treated group; H3C: hypoxic groups were treated with crocin. B1, B2, and B3; three blocks of training stage in Morris water maze test. ***p < .001, ****p < .0001 as compared to the sham group. ×××*p* < .001, ××××*p* < .0001 in comparison with the CC group and @@*p* < .01, @@@*p* < .001 as compared to the H3C rats. Data presented as mean ± *SEM* (*n* = 7)

Rats in all groups showed a significant reduction in the TD and LT to reach the hidden platform in third block of MWM showing memory acquisition. The results showed that the TD and TL to reach the hidden platform was significantly higher in H3 compared to the other hypoxia and sham groups (*p* < .0001) (Figure 3a,b). Crocin treatment in H3 group (H3C) resulted a significant reduction of TD and TL compared to hypoxia (H3) group (*p* = .0005), and the difference between H3C, CC, and sham groups was not significant (Figure 4a,b).

#### 3.1.2 Swimming speed and traveled distance in the target quadrant

Like the learning test, in the probe stage, we initially compared three hypoxia groups with each other and sham group. After that, the H3 group was assessed and compared with sham, crocin, and H3C groups.

**FIGURE 5:**
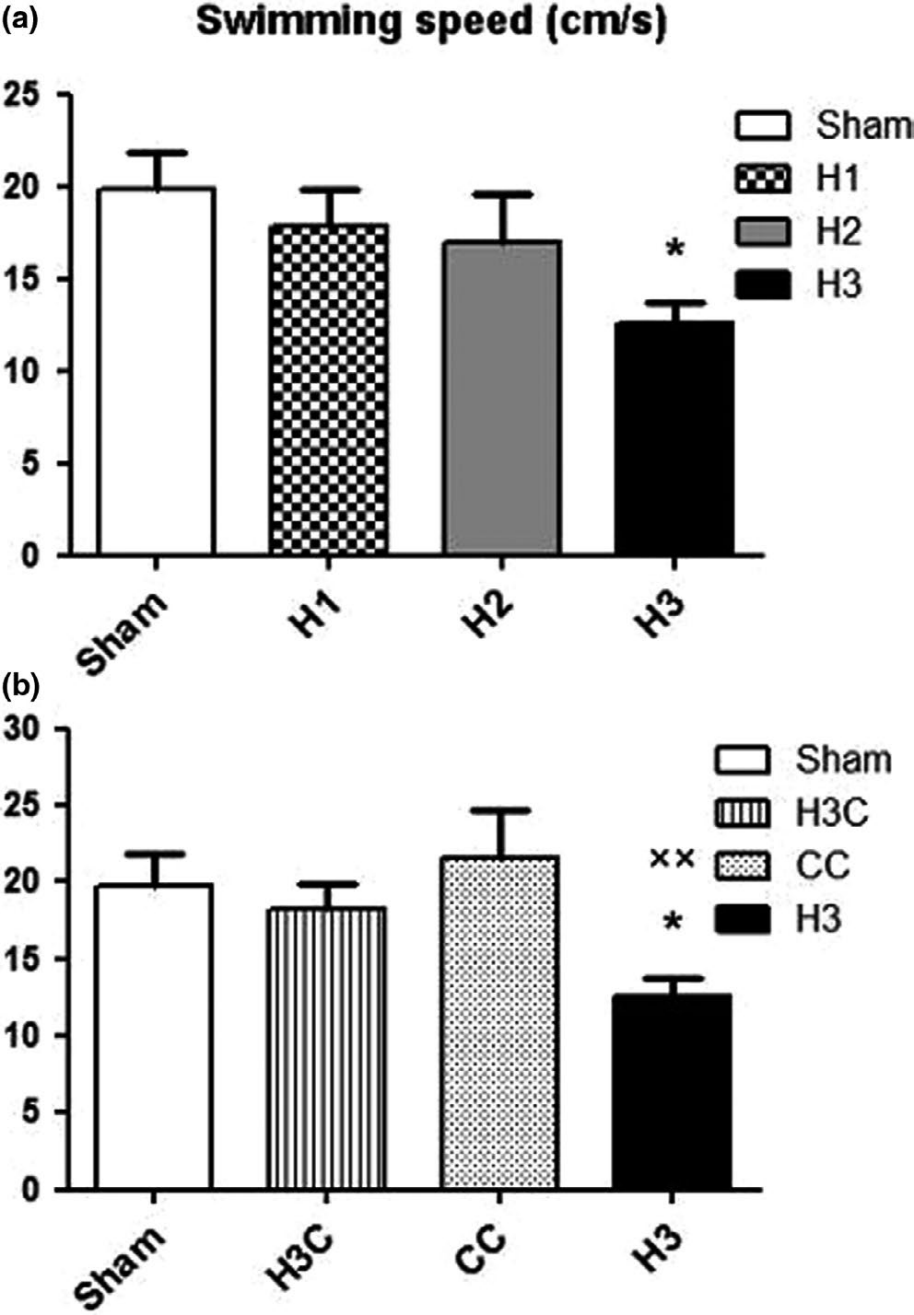
Swimming speed in correct quadrant was significantly decreased in H3 group compared to the sham group (a) crocin treatment had significant effect on the speed of swimming compared to the H3 group and the difference between H3C and sham groups were not significant (b). H3: hypoxia 3; CC: control crocin treated group; H3C: hypoxic groups were treated with crocin. *p < .05 as compared to the sham group. ××*p* < .01 in comparison with the CC group. Data presented as mean ± *SEM* (*n* = 7)

**FIGURE 6:**
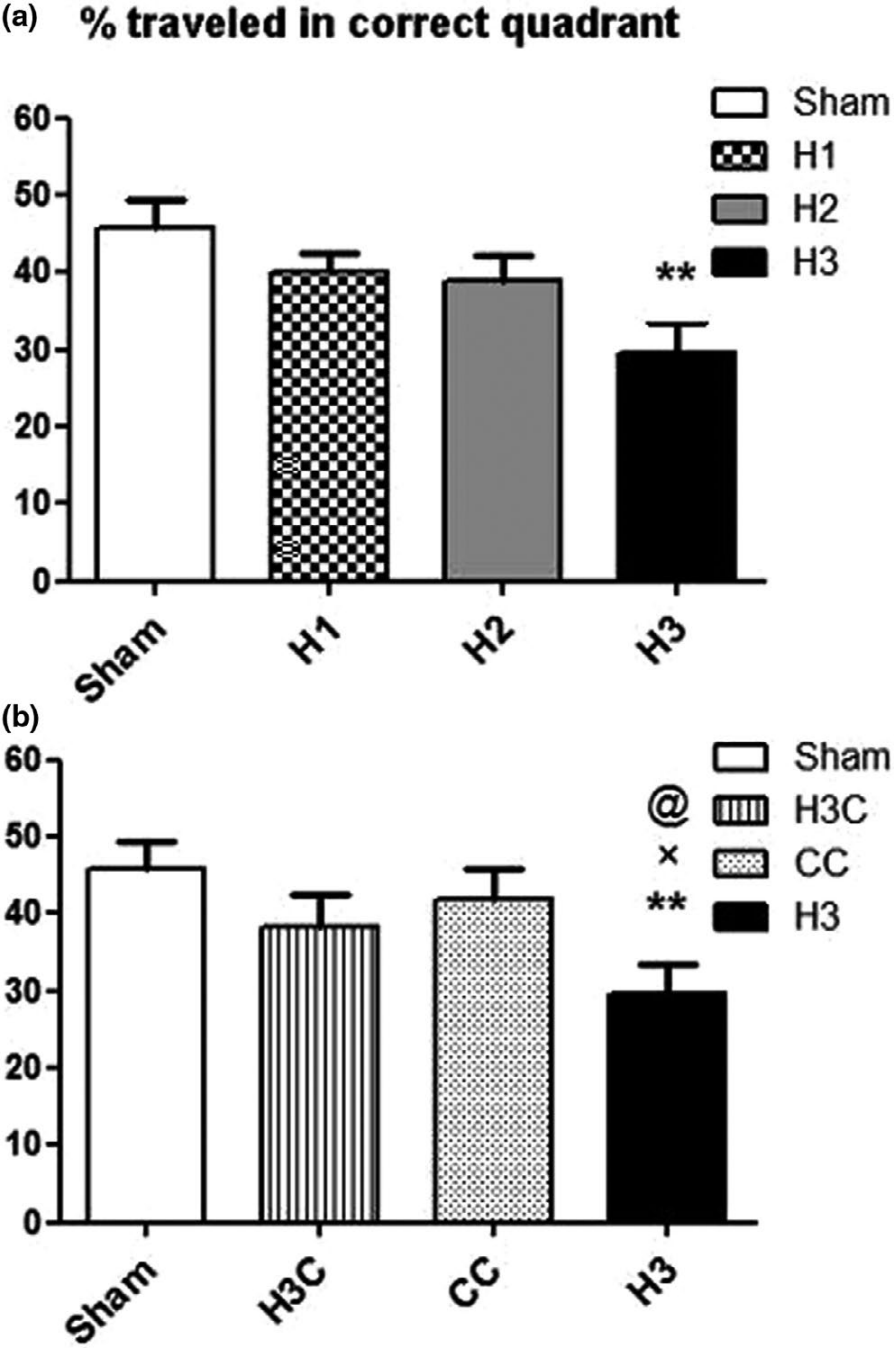
The percent of traveled distance in correct quadrant was significantly decreased in H3 group compared to the sham group (a). Traveled distance also decreased in H3 in comparison with CC and H3C groups (b), and the H3C group did not show any significantly difference with CC and sham groups (c). H3: hypoxia 3; CC: control crocin treated group; H3C: hypoxic groups were treated with crocin. **p < .01 as compared to the sham group. ×*p* < .05 in comparison with the CC group. @*p* < .05 as compared to the H3C rats. Data presented as mean ± *SEM* (*n* = 7)

The mean percentage (%) of SS in correct quadrant was significantly decreased in H3 group compared to the sham group (*p* = .0283) (Figure 5a). But the percentage of SS in crocin treated group was significantly increased compared to the hypoxia (H3) (*p* = .0026) (Figure 5b). Our results also showed that induction of hypoxia (H3) caused a significant TD decrease in the correct quadrant compared to the sham (*p* = .0051) (Figure 6a) but crocin treatment after prenatal hypoxia induction resulted a significant increasing of TD (*p* = .0137) (Figure 6b).

### 3.2 qRT‐PCR analysis

#### 3.2.1 Effect of crocin treatment on mRNA level of HIF‐1α in brain of neonates under maternal hypoxia

**FIGURE 7:**
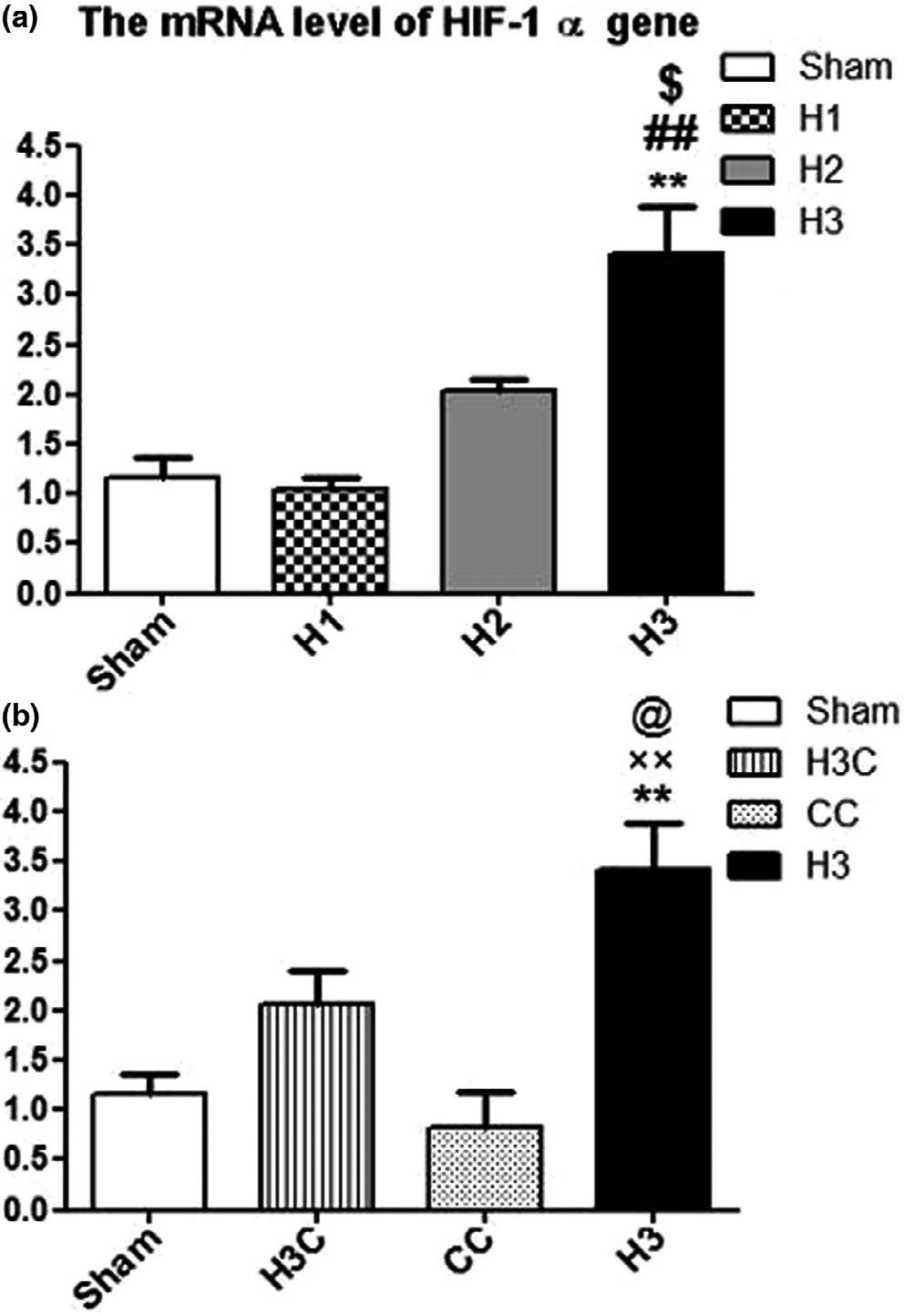
Comparison of the mRNA level of HIF‐1α gene expression. The mRNA level of HIF‐1α was significantly increased in H3 group compared to the other hypoxia groups (H1 and H2) and sham (a). H3: hypoxia 3; CC: control crocin treated group; H3C: hypoxic groups were treated with crocin. **p < 0.01compared to the sham and ##*p* < .01 compared to the H1 group. $ was also show the significant difference (*p* < .05) between H3 and H2. ××*p* < .05 in comparison with the CC group. @*p* < .05 as compared to the H3C rats. Data presented as mean ± *SEM* (*n* = 5)

Comparison the HIF‐1α mRNA levels between different experimental groups showed that the mRNA levels of HIF‐1α in H3 groups was significantly increased compared to the sham, (*p* = .0019), H1 (*p* = .0034) and H2 (*p* = .0240) while induction of hypoxia had no significant effect on expression of HIF‐1α in H1 group compared to the sham group (*p* > .05) (Figure 7a). Crocin treatment could attenuate the increased levels of HIF‐1α mRNA in H3C groups compared to hypoxia (*p* = .0291), untreated groups (Figure 7b).

#### 3.2.2 Effect of crocin treatment on mRNA level of BACE1 in brain of neonates under maternal hypoxia

**FIGURE 8:**
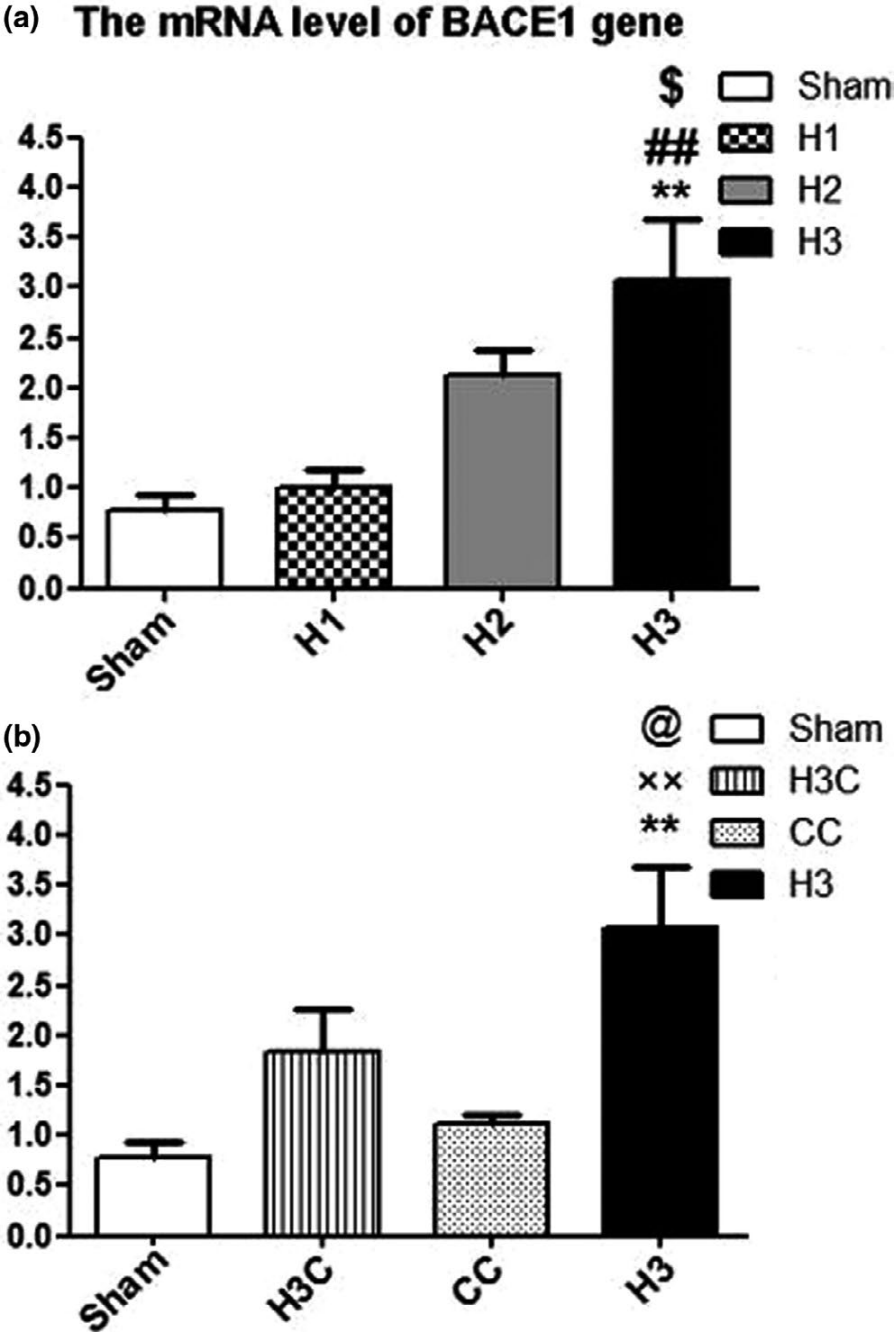
Comparison of the mRNA level of BACE1 gene expression. The mRNA level of BACE1 was significantly increased in H3 group compared to the other hypoxia groups (H1 and H2) and sham (a). **p < 0.01compared to the sham and ##*p* < .01 compared to the H1 group. $ was also show the significant difference (*p* < .05) between H3 and H2. ××*p* < .05 in comparison with the CC group. @*p* < .05 as compared to the H3C rats (b). H3: hypoxia 3; CC: control crocin treated group; H3C: hypoxic groups were treated with crocin. Data presented as mean ± *SEM* (*n* = 5)

As shown in Figure 8, the mRNA level of BACE1 was significantly increased in H3 groups compared to the Sham (*p* = .0083), H1 (*p* = .0045) and H2 (*p* = .0213) (Figure 8a). Crocin treatment could attenuate the increased levels of BACE1 mRNA H3C group compared to hypoxia (*p* = .0045), untreated groups (Figure 8b).

## 4 DISCUSSION

Our results showed that maternal hypoxia induction caused memory deficit in the MWM test and the most destructive effect was related to the hypoxia with 93% N_2_ and 7% O_2_ intensity for 3 hr. So that, SS decreased while time to find the hidden platform increased in the H3 group, and this decreasing in SS can be effected the latency time to find the platform. Ischemia or hypoxia during pregnancy results in embryo hypoxia and are associated with adverse outcomes for newborn infants such as memory impairment and susceptibility to neurodegenerative disease after birth (Zhang et al., 2013). Pregnancy hypoxia not only increases abortion risk but also results in developmental and neuronal disorder later in life due to neuroinflammation, neuronal apoptosis, oxidative stress, and molecular changes related to protein misfolding in brain (Mulder et al., 2002).

In the present study, the protective effect of crocin against cognitive memory impairment and molecular alterations related to β‐amyloid formation and hypoxia induced by maternal hypoxia were studied in rat offspring after birth. A significant loss of learning and spatial memory have been found in neonates transgenic rats (APPSwe /PS1A246E) when exposed to hypoxia by putting them in a hypobaric chamber between 7th and 20th days of pregnancy (Zhang et al., 2013). It has also been reported an increase in β‐amyloid precursor protein level, a lower level of Neprilysin, β‐amyloid degrading enzyme, and increase accumulation of β amyloid in prenatal hypoxic rat's brain (Tab aton & Tamagno, 2007; Zhang et al., 2007). In our study, results of Morris water maze task indicated that hypoxia reduce the mean of traveled distance in the target quadrant at the probe stage and this decreases dependent to the intensity and duration of hypoxia. Although crocin treatment increased traveled distance in the target quadrant at the probe stage.

A lot of studies showed that total protein and activity of BACE1 increase in area of brain which affected by Alzheimer disease and indicated that abnormal activity of BACE1 had a functional role in Alzheimer pathogenesis (Das & Yan, 2017). Wen and his colleague's results indicated significant increase in activity and BACE1 expression in rats which were affected by transient brain ischemia (Wen et al., 2004). Our results showed that expression of β‐amyloid associated genes (BACE1 and HIF‐1α) increase with intensity and duration of hypoxia and crocin treatment improved memory by reducing the expression of these genes. Results of the researchers in this regard showed that hypoxia increase expression and activation of this enzyme by upregulation of BACE1 mRNA. Increased action of BACE1 following hypoxia increases β‐amyloid production from β‐amyloid precursor protein (Jellinger & Attems, 2005). Hypoxia increases BACE1 enzyme's activity by expression of HIF‐1α, and there is a direct relation between expression of HIF‐1α gene and BACE1 enzymes activity (Zhang et al., 2007). In one study, following β‐amyloid injection and induction of an Alzheimer model in rats, IP injection of crocin significantly improved spatial memory clues in Morris water maze task such as escape time, traveled distance, and time spent in the target quarter compared to the group received β‐amyloid (Papandreou et al., 2006). Also indicated that crocin inhibits apoptosis induced by β‐amyloid, this inhibition possibly related to its antioxidant properties (Papandreou et al., 2006). Hosseinzadeh et al. (2012) investigated crocin effect and saffron extract on cognition impairment and memory associated with ischemia by Morris water maze and have shown that IP injection of crocin by dosage 5–25 mg/kg and saffron extract (50–250 mg/kg) in ischemic rats can recover or improve memory and cognition impairments. In addition to all mentioned above, another reason for AD is imbalance between the generation of free radicals and antioxidants. Because of high consumption of O_2_, low levels of polyunsaturated fatty acids, and low levels of antioxidants, the brain is so susceptible to oxidative stress (Floyd & Hensley, 2009). Aβ and oxidative stress are related to each other, Aβ accumulation induces oxidative stress in vivo and in vitro and the production of Aβ increases by oxidants (Floyd & Hensley, 2009; Paola et al., 2000). Also, there is a significant relation between BACE1 activity and oxidative markers in sporadic form of AD brain tissue (Borghi et al., 2007), because expression and activity of BACE1 are increased by oxidants (Tamagno, Bardini, et al., 2012; Tamagno, Guglielmotto, et al., 2012). Chandel et al. suggested that the source of ROS in the hypoxic response is mitochondria and it is essential to activate HIF‐1α. It has been shown that antioxidants inverse the activation of hypoxia‐induced HIF‐1α (Aminova et al., 2008).

HIF‐1α binds to the BACE1 promoter and regulates its gene expression. As mentioned before, overexpression of HIF‐1α following hypoxia can increase BACE1 mRNA, and consequently led to β‐amyloid production from β‐amyloid precursor protein (Zhang et al., 2007). Antioxidant property of saffron and crocin can be considered as a possible mechanism for protecting neurons in the CNS from oxidative damage caused by hypoxia‐induced memory impairment because saffron and crocin destroy and eliminate free radicals like ROS (Naghizadeh et al., 2010; Zhang et al., 2007). In this regard, study of Ghadrdoost et al. (2011) showed that saffron extract by its antioxidant property decrease hippocampus oxidative stress induced by chronic stress.

In summary, increased level of ROS following hypoxia will activate PHDs, ERK, PKB/Akt, and p38MAPK pathways, so because of activation of these pathways, HIF‐1α subsequently upregulates and binds to BACE1 promotor, this event results in increases of β amyloid production from APP. Therefore, the probable mechanism for effect of crocin to improve hypoxia condition is that, following injection of crocin after hypoxia, it will neutralize produced ROS and prevents the activation of PHDs, ERK, PKB/Akt, and p38MAPK pathways, so that HIF‐1α amount will not increase and consequently BACE1 activity will reduced. This process leads to reduction of Aβ production, accumulation, and memory improvement.

According to our results, transcription of BACE1 mRNA via increasing HIF‐1α expression in offspring was increased after maternal hypoxia. Following that the molecular mechanism run to facilitate β‐amyloid formation and cognitive impairment, in this regard hypoxia with 93% N_2_ and 7% O_2_ intensities for 3 hr had the most destructive effect on cognitive behavior in rat's offspring and crocin had protective effect by inhibiting the molecular pathway to β‐amyloid formation and cognitive impairment.

## CONFLICT OF INTEREST

The authors declare that they have no conflict of interest.

## AUTHOR CONTRIBUTION

Zohreh Ghotbeddin participated in study design, data collection, and evaluation and contributed behavioral experiments. Mohammad Reza Tabandeh participated in study design, data collection, evaluation, conduction of molecular experiments and qRT‐PCR analysis, and responsible for overall supervision. Mahdi Pourmahdi Borujeni participated in data and statistical analysis. Fahimeh Fahimi Truski, Reza Zalaki Ghorbani Pour, and Leila Tabrizian contributed to all experimental work. All authors performed editing and approving the final version of this paper for submission, also participated in the finalization of the manuscript, and approved the final draft.

### PEER REVIEW

The peer review history for this article is available at https://publons.com/publon/10.1002/brb3.2078.

## Data Availability

The data that support the findings of this study are available from the corresponding author upon reasonable request.

## References

[brb32078-bib-0001] Aminova, L. R. , Siddiq, A. , & Ratan, R. R. (2008). Antioxidants HIF prolyl hydroxylase inhibitors or short interfering RNAs to BNIP3 or PUMA, can prevent prodeath effects of the transcriptional activator, HIF‐1α, in a mouse hippocampal neuronal line. Antioxidants & Redox Signaling, 10(12), 1989–1998. 10.1089/ars.2008.2039 18774900 PMC2612757

[brb32078-bib-0002] Borghi, R. , Patriarca, S. , Traverso, N. , Piccini, A. , Storace, D. , & Garuti, A. (2007). The increased activity of BACE1 correlates with oxidative stress in Alzheimer's disease. Neurobiology of Aging, 28(7), 1009–1014. 10.1016/j.neurobiolaging.2006.05.004 16769154

[brb32078-bib-0003] Bustin, S. A. , Benes, V. , Garson, J. A. , Hellemans, J. , Huggett, J. , Kubista, M. , Mueller, R. , Nolan, T. , Pfaffl, M. W. , Shipley, G. L. , Vandesompele, J. , & Wittwer, C. T. (2009). The MIQE guidelines: Minimum information for publication of quantitative real‐time PCR experiments. Clinical Chemistry, 55(4), 611–622. 10.1373/clinchem.2008.112797 19246619

[brb32078-bib-0004] Das, B. , & Yan, R. (2017). Role of BACE1 in Alzheimer's synaptic function. Translational Neurodegeneration, 6(1), 23. 10.1186/s40035-017-0093-5 28855981 PMC5575945

[brb32078-bib-0005] Depino, A. (2015). Early prenatal exposure to LPS results in anxiety‐and depression‐related behaviors in adulthood. Neuroscience, 299, 56–65. 10.1016/j.neuroscience.2015.04.065 25943476

[brb32078-bib-0006] Desmond, D. W. , Moroney, J. T. , Sano, M. , & Stern, Y. (2002). Incidence of dementia after ischemic stroke: Results of a longitudinal study. Stroke, 33(9), 2254–2262. 10.1161/01.STR.0000028235.91778.95 12215596

[brb32078-bib-0007] Floyd, R. A. , & Hensley, K. (2009). Oxidative stress in brain aging: Implications for therapeutics of neurodegenerative diseases. Neurobiology of Aging, 23(5), 795–807. 10.1016/S0197-4580(02)00019-2 12392783

[brb32078-bib-0008] Frick, K. M. , Stillner, E. T. , & Berger‐Sweeney, J. (2000). Mice are not little rats: Species differences in a one‐day water maze task. NeuroReport, 11(16), 3461–3465. 10.1097/00001756-200011090-00013 11095500

[brb32078-bib-0009] Georgiadou, G. , Grivas, V. , Tarantilis, P. A. , & Pitsikas, N. (2014). Crocins, the active constituents of *Crocus sativus* L., counteracted ketamine–induced behavioural deficits in rats. Psychopharmacology (Berlin), 231(4), 717–726 24096536

[brb32078-bib-0010] Getahun, D. , Rhoads, G. G. , Demissie, K. , Lu, S. E. , Quinn, V. P. , Fassett, M. J. , Wing, D. A. , & Jacobsen, S. J. (2013). In utero exposure to ischemic‐hypoxic conditions and attention‐deficit/hyperactivity disorder. Pediatrics, 131(1), e53–e61. 10.1542/peds.2012-1298 23230063

[brb32078-bib-0011] Ghadrdoost, B. , Vafaei, A. A. , Rashidy‐Pour, A. , Hajisoltani, R. , Bandegi, A. R. , Motamedi, F. , Haghighi, S. , Sameni, H. R. , & Pahlvan, S. (2011). Protective effects of saffron extract and its active constituent crocin against oxidative stress and spatial learning and memory deficits induced by chronic stress in rats. European Journal of Pharmacology, 667(1–3), 222–229. 10.1016/j.ejphar.2011.05.012 21616066

[brb32078-bib-0012] Ghotbeddin, Z. , Tabandeh, M. R. , Pourmahdi Borujeni, M. , Fahimi Truski, F. , & Tabrizian, L. (2018). Study the effect of crocin in three maternal hypoxia protocols with different oxygen intensities on motor activity and balance in rat offspring. Acta Neurologica Belgica, 120, 155–161 29882009 10.1007/s13760-018-0953-5

[brb32078-bib-0013] Golan, H. , Kashtutsky, I. , Hallak, M. , Sorokin, Y. , & Huleihel, M. (2004). Maternal hypoxia during pregnancy delays the development of motor reflexes in newborn mice. Developmental Neuroscience, 26(1), 24–29. 10.1159/000080708 15509895

[brb32078-bib-0014] Hosseinzadeh, H. , Sadeghnia, H. R. , Ghaeni, F. A. , Motamedshariaty, V. S. , & Mohajeri, S. A. (2012). Effects of saffron (*Crocus sativus* L.) and its active constituent, crocin, on recognition and spatial memory after chronic cerebral hypoperfusion in rats. Phytotherapy Research, 26(3), 381–386 21774008 10.1002/ptr.3566

[brb32078-bib-0015] Huang, H. C. , & Jiang, Z. F. (2009). Accumulated amyloid‐β peptide and hyperphosphorylated tau protein: Relationship and links in Alzheimer's disease. Journal of Alzheimer's Disease, 16(1), 15–27. 10.3233/JAD-2009-0960 19158417

[brb32078-bib-0016] Jellinger, K. A. , & Attems, J. (2005). Prevalence and pathogenic role of cerebrovascular lesions in Alzheimer disease. Journal of Neurological Science, 229–230, 37–41. 10.1016/j.jns.2004.11.018 15760617

[brb32078-bib-0017] Koeppen, B. , & Stanton, B. A. (2009). Berne and levy physiology. Elsevier.

[brb32078-bib-0018] Mondragón‐Rodríguez, S. , Perry, G. , Zhu, X. , & Boehm, J. (2012). Amyloid beta and tau proteins as therapeutic targets for Alzheimer's disease treatment: Rethinking the current strategy. International Journal of Alzheimer's Disease, 2012, 1–7. 10.1155/2012/630182 PMC3310047 22482074

[brb32078-bib-0019] Mulder, E. J. , De Medina, P. R. , Huizink, A. C. , Van den Bergh, B. R. , Buitelaar, J. K. , & Visser, G. H. (2002). Prenatal maternal stress: Effects on pregnancy and the (unborn) child. Early Human Development, 70(1–2), 3–14. 10.1016/S0378-3782(02)00075-0 12441200

[brb32078-bib-0020] Naghizadeh, B. , Mansouri, M. , Ghorbanzadeh, B. , Farbood, Y. , & Sarkaki, A. (2013). Protective effects of oral crocin against intracerebroventricular streptozotocin‐induced spatial memory deficit and oxidative stress in rats. Phytomedicine, 20(6), 537–542. 10.1016/j.phymed.2012.12.019 23351962

[brb32078-bib-0021] Naghizadeh, B. , Mansouri, S. M. T. , & Mashhadian, N. V. (2010). Crocin attenuates cisplatin‐induced renal oxidative stress in rats. Food and Chemical Toxicology, 48(10), 2650–2655. 10.1016/j.fct.2010.06.035 20600529

[brb32078-bib-0022] Nalivaeva, N. N. , Fisk, L. , Kochkina, E. G. , Plesneva, S. A. , Zhuravin, I. A. , Babusikova, E. , Dobrota, D. , & Turner, A. J. (2004). Effect of hypoxia/ischemia and hypoxic preconditioning/reperfusion on expression of some amyloid‐degrading enzymes. Annals of the New York Academy of Science, 1035, 21–33. 10.1196/annals.1332.002 15681798

[brb32078-bib-0023] Nisbet, R. M. , Polanco, J. C. , Ittner, L. M. , & Götz, J. (2015). Tau aggregation and its interplay with amyloid‐β. Acta Neuropathologica, 129(2), 207–220. 10.1007/s00401-014-1371-2 25492702 PMC4305093

[brb32078-bib-0024] Paola, D. , Domenicott, C. , Nitti, M. , Vitali, A. , Borghi, R. , Cottalasso, D. , Zaccheo, D. , Odetti, P. , Strocchi, P. , Marinari, U. M. , Tabaton, M. , & Pronzato, M. A. (2000). Oxidative stress induces increase in intracellular amyloid β‐protein production and selective activation of βI and βII PKCs in NT2 cells. Biochemical and Biophysical Research Communications, 268(2), 642–646. 10.1006/bbrc.2000.2164 10679257

[brb32078-bib-0025] Papandreou, M. A. , Kanakis, C. D. , Polissiou, M. G. , Efthimiopoulos, S. , Cordopatis, P. , & Margarity, M. (2006). Inhibitory activity on amyloid‐β aggregation and antioxidant properties of *Crocus sativus* stigmas extract and its crocin constituents. Journal of Agricultural & Food Chemistry, 54(23), 8762–8768. 17090119 10.1021/jf061932a

[brb32078-bib-0026] Perlman, J. M. (2007). Pathogenesis of hypoxic‐ischemic brain injury. Journal of Prenatology, 27, 39–46. 10.1038/sj.jp.7211716

[brb32078-bib-0027] Semenza, G. L. (1998). Hypoxia‐inducible factor 1: Master regulator of O2 homeostasis. Current Opinion in Genetics & Development, 8(5), 588–594. 10.1016/S0959-437X(98)80016-6 9794818

[brb32078-bib-0028] Tabaton, M. , & Tamagno, E. (2007). The molecular link between β‐ and γ‐secretase activity on the amyloid β precursor protein. Cellular and Molecular Life Sciences, 64(17), 2211–2218. 10.1007/s00018-007-7219-3 17604999 PMC11136381

[brb32078-bib-0029] Tamagno, E. , Bardini, P. , Obbili, A. , Vitali, A. , Borghi, R. , & Zaccheo, D. (2012). Oxidative stress increases expression and activity of BACE in NT2 neurons. Neurobiology of Diseases, 10(3), 279–288. 10.1006/nbdi.2002.0515 12270690

[brb32078-bib-0030] Tamagno, E. , Guglielmotto, M. , Monteleone, D. , & Tabaton, M. (2012). Amyloid‐β production: Major link between oxidative stress and BACE1. Neurotoxicity Research, 22(3), 208–219. 10.1007/s12640-011-9283-6 22002808

[brb32078-bib-0031] Venugopal, C. , Demos, C. M. , Rao, K. S. , Pappolla, M. A. , & Sambamurti, K. (2006). Beta‐secretase: Structure, function, and evolution. CNS & Neurological Disorders ‐ Drug Targets, 7(3), 278–294. 10.2174/187152708784936626 PMC2921875 18673212

[brb32078-bib-0032] Wang, X. , Zhu, M. , Hjorth, E. , CortésToro, V. , Eyjolfsdottir, H. , Graff, C. , Nennesmo, I. , Palmblad, J. , Eriksdotter, M. , Sambamurti, K. , Fitzgerald, J. M. , Serhan, C. N. , Granholm, A.‐C. , & Schultzberg, M. (2015). Resolution of inflammation is altered in Alzheimer's disease. Alzheimer's & Dementia, 11(1), 40–50. 10.1016/j.jalz.2013.12.024 PMC4275415 24530025

[brb32078-bib-0033] Wen, Y. , Onyewuchi, O. , Yang, S. , Liu, R. , & Simpkins, J. W. (2004). Increased β‐secretase activity and expression in rats following transient cerebral ischemia. Brain Research, 1009(1–2), 1–8. 15120577 10.1016/j.brainres.2003.09.086

[brb32078-bib-0034] Wenger, R. H. , & Gassmann, M. (1997). Oxygen(es) and the hypoxia‐inducible factor‐1. Biological Chemistry, 378(7), 609–616. 9278140

[brb32078-bib-0035] Zhang, X. , Li, L. , Zhang, X. , Xie, W. , Li, L. , & Yang, D. (2013). Prenatal hypoxia may aggravate the cognitive impairment and Alzheimer's disease neuropathology in APPSwe/PS1A246E transgenic mice. Neurobiology of Aging, 34(3), 663–678. 10.1016/j.neurobiolaging.2012.06.012 22795785

[brb32078-bib-0036] Zhang, X. , Zhou, K. , Wang, R. , Cui, J. , Lipton, S. A. , Liao, F. , Xu, H. , & Zhang, Y.‐W. (2007). Hypoxia‐inducible factor 1α (HIF‐1α)‐mediated hypoxia increases BACE1 expression and β‐amyloid generation. Journal of Biological Chemistry, 282(15), 10873–10880. 10.1074/jbc.M608856200 17303576

